# A multi-disciplinary comparison of great ape gut microbiota in a central African forest and European zoo

**DOI:** 10.1038/s41598-020-75847-3

**Published:** 2020-11-05

**Authors:** Victor Narat, Katherine R. Amato, Noémie Ranger, Maud Salmona, Séverine Mercier-Delarue, Stephanie Rupp, Philippe Ambata, Richard Njouom, François Simon, Tamara Giles-Vernick, Jérôme LeGoff

**Affiliations:** 1 Eco-anthropologie, UMR7206 CNRS/MNHN/Université de Paris, Site du Musée de L’Homme, Paris, France; 2 Institut Pasteur, Anthropology and Ecology of Disease Emergence Unit, Paris, France; 3 Department of Anthropology, Northwestern University, Evanston, USA; 4 Humans and the Microbiome, CIFAR, Toronto, Canada; 5 Université de Paris, Equipe INSIGHT, Inserm U976, 75010 Paris, France; 6 Département des Agents Infectieux, Virologie et Greffes, AP-HP, Hôpital Saint-Louis, 75010 Paris, France; 7 Department of Anthropology, City University of New York – Lehman College, New York, NY USA; 8 Ministry of Agriculture and Rural Development, Yaounde, Cameroon; 9 Centre Pasteur du Cameroun, Yaounde, Cameroon

**Keywords:** Microbiome, Environmental impact

## Abstract

Comparisons of mammalian gut microbiota across different environmental conditions shed light on the diversity and composition of gut bacteriome and suggest consequences for human and animal health. Gut bacteriome comparisons across different environments diverge in their results, showing no generalizable patterns linking habitat and dietary degradation with bacterial diversity. The challenge in drawing general conclusions from such studies lies in the broad terms describing diverse habitats (“wild”, “captive”, “pristine”). We conducted 16S ribosomal RNA gene sequencing to characterize intestinal microbiota of free-ranging sympatric chimpanzees and gorillas in southeastern Cameroon and sympatric chimpanzees and gorillas in a European zoo. We conducted participant-observation and semi-structured interviews among people living near these great apes to understand better their feeding habits and habitats. Unexpectedly, bacterial diversity (ASV, Faith PD and Shannon) was higher among zoo gorillas than among those in the Cameroonian forest, but zoo and Cameroonian chimpanzees showed no difference. Phylogeny was a strong driver of species-specific microbial composition. Surprisingly, zoo gorilla microbiota more closely resembled that of zoo chimpanzees than of Cameroonian gorillas. Zoo living conditions and dietary similarities may explain these results. We encourage multidisciplinary approach integrating environmental sampling and anthropological evaluation to characterize better diverse environmental conditions of such investigations.

## Introduction

Over the last decade, numerous studies have demonstrated the importance of environmental changes on the mammalian gut microbiome, which is strongly associated with host metabolic, immune, and neurological functions^1^. Broad-ranging influences, including host genetics, living conditions, diet, stress, and antibiotic use can affect gut microbial diversity^2–5^. Among these influences, diet and living conditions have been evaluated for human and animal populations, entailing significant effects on gut microbiota and consequences for human and animal health. Adverse microbial profile shifts, for instance, have been associated with dysbiosis and wide-ranging diseases among human beings, from obesity to pediatric environmental enteropathy, and from autism to asthma^2,6,7^.

Outside of laboratory conditions, disentangling the effects of living and dietary conditions on gut microbial composition from other influences remains a complex question. In humans, such questions have catalyzed multiple studies comparing environmental and gut microbiota between “westernized” and “rural” peoples^8–10^. Among other mammalian populations, degradation in habitat quality affects the diversity of available flora and fauna for consumption, and in some cases, is associated with declines in microbial gut composition^11–14^. Microbiome studies of nonhuman primates (NHPs) can offer rich insight for humans and other mammalian life: NHP health is essential for species and environmental conservation, and these primates display high diversity and adaptability across ecological niches, complex social organization, wide geographic distribution, and evolutionary proximity to human beings^15–17^. Yet relatively few NHP microbiome studies have been conducted, and even fewer on great apes, whose genetic proximity, adaptability across multiple ecological zones and to changing alimentary regimens, and co-speciation of some gut bacteria render them a useful model^18–20^. Such studies are also valuable because the conservation status of great apes is threatened^11,21,22^.

Thus far, investigations have compared gut microbiota of NHP populations over different time scales and under multiple conditions, with varied results. One comparison across 18 NHP species, for instance, revealed that host phylogeny constitutes a primary influence on bacterial diversity^23^. Studies along gradients of habitat degradation have revealed that certain NHP species inhabiting more disturbed sites had decreased gut microbial diversity^11,24,25^. Four other NHP species in Uganda displayed no association between gut bacterial diversity and habitat degradation, although that study did not use the same methods to categorize habitat degradation and to condition feces as other investigations^12^. Controlled comparisons between NHP gut ecologies under “wild” or “pristine” and “captive” conditions show a decrease in alpha diversity among more than 20 NHP species under zoo conditions^2,26–28^. Some analyses explicitly argue that reduced alpha diversity among captive NHP species suggests a similar pattern to that of “westernized (human) societies”^2^.

These varied conclusions suggest that the labels used to describe conditions— “wild”, “captive”, “westernized”, “rural”, “pristine”, “disturbed” – may obscure more than they reveal. “Wild” NHPs suggests that they live in isolation from human presence; yet they have shared habitats with humans for millennia and have adapted to anthropogenically altered terrains^29–32^. Similarly, “captive” NHPs can live under highly variable conditions. Such terms thus mask important differences in microbial exposures. One investigation of nine colobus species housed in five different zoos underscored the importance of diet on gut microbiota, but also acknowledged that uninvestigated environmental features of these zoos could also influence gut microbial composition^33^. Comparisons of gut microbiota diversity therefore require better characterization and analysis of diverse environmental and dietary conditions in which these investigations are conducted^34^.

In the present study, we conducted 16S ribosomal RNA gene sequencing to characterize the intestinal microbiota of free-ranging sympatric chimpanzees (*Pan troglodytes troglodytes*) and gorillas (*Gorilla gorilla gorilla*) in the dense forests of southeastern Cameroon and of sympatric chimpanzees (*P.t. verus*) and gorillas (*G.g. gorilla*) living in a European zoo. We hypothesized that zoo conditions would occasion a decline in gut bacterial diversity. Rather than reducing this comparison to “wild” and “captive” conditions, we combined our gut composition analysis with anthropological participant-observation and targeted semi-structured interviews among people in close proximity to these animals (zookeepers in the zoo, local populations in Cameroon), complemented by published studies about central African great ape diets, to understand better their feeding habits and living conditions.

## Results

### Characterization of environment of great apes in southeastern Cameroon and the zoo setting

Table 1 summarizes characteristics of the two study sites, comparing animal group size, environment, contacts with other NHPs and humans, and diet. Below we signal the most salient commonalities and differences found in environment, diet, and inter-species contacts, which can influence microbiota.

**Table 1:** Environment, diet and interspecies contacts for gorillas and chimpanzees in southeastern Cameroon and in the European zoo.

	Study site		Chimpanzees *Pan t. troglodytes*	Gorillas *Gorilla g. gorilla*	Sources
Group size	Cameroon		9	9–12	Number of nests during feces sampling
	Zoo		4 males and 3 females	1 adult male, 4 females, 5 infants/juveniles	Direct observation
Environment	Cameroon	*Density*	0.17 ind/km^2^	2.5–3 ind/km^2^	^35^
		*Habitat preferences*	Mainly in mixed mature forests with closed canopy	Mainly in open canopy forests with dense herbaceous vegetation	^36–39^
		*% of land cover represented by rivers*	~ 0.1% of the area		^39^
	Zoo	*Indoor enclosure*	215 m^2^ Straw and wood chips on the ground Various horizontal and vertical structures	115 m^2^ Straw and wood chips on the ground Various horizontal and vertical structures	Interviews with zoo zookeepers and direct observations
		*Outdoor enclosure*	7000 m^2^ Herbaceous vegetation: herbs, iris, water daffodils, bramble Trees: goat willow, wild cherry, hornbeam, oak, chestnut Water channel around the island	5000 m^2^ Herbaceous vegetation: herbs, bramble, water daffodil, rushes Trees: oak, maple, chestnut Water channel around the island	Interviews with zoo zookeepers and direct observations
Diet	Cameroon	*Diet composition*	Mainly fruits Leaves, shoots, pith Occasionally mammals, insects and honey 132 plant species	Mainly leaves, shoots, pith and roots but increasing of fruit consumption when fruit availability is higher Occasionally insects 150–180 plant species	^40–44^
		*Geophagy*	Regular/Frequent	Frequent	^43,45^
		*Charcoal consumption*	Ash consumption reported once	Not reported	^46^
	Zoo	*Meal organization*	4–7/day. All individuals eat together	4–7/day. Separate: silverback eats alone; small groups composed of adult female and her offspring	Interviews with zoo zookeepers and direct observations
			~ 80% vegetable matter and 20% fruits 15–20 different food species/preparation		Interviews with zoo zookeepers and direct observations
		*Food provided—Morning*	Salad, carrot, apple, "Old World Monkey" chow, Rice and one fruit (banana, orange, kiwi, pear …)	Salad, celery, turnip, fennel, apple, cabbages, “Old World Monkey” chow Branches (mainly hazelnut) for bark, fruits, leaves	Interviews with zoo zookeepers and direct observations
		*Food provided—Day time*	Vegetables only	Cucumber, endive, carrot	Interviews with zoo zookeepers and direct observations
		*Food provided—Evening*	Salad, apple, carrot, bell pepper, 1 fruit (banana, orange, kiwi, pear …)	Salad, leek, tomato, bell pepper, broccoli, 1 fruit (varied) Locally-made biscuit (wheat flour, soy flour, oatmeal, vitamins, salt) Large branches (bark)	Interviews with zoo zookeepers and direct observations
		*Food provided—1 times/week*	Cold weather: Tea, vegetable broth Hot weather: grenadine syrup Hard boiled egg Cooked meat (chicken, turkey)	Almonds, other nuts and seeds	Interviews with zoo zookeepers and direct observations
		*Food provided—Occasional*	Seeds (wheat, maize, sunflower, almond, peanuts, walnuts, oatmeal) Flour worms Boiled potatoes Bread	During winter: enrichment with wheat flour or honey in enclosure	Interviews with zoo zookeepers and direct observations
		*Opportunistic consumption from the island*	Spring: Wild cherry (young leaves, fruits), Hornbeam (bark) Autumn: Oak (Fruits), Chestnut (Fruits) All seasons: iris (leaves) Mixed piece of fruits with straw, herbs or dried leaves to masticate as a kind of “chewing gum” Geophagy (rare)	All seasons: herbs, rushes (leaves), water daffodil (leaves), bramble (leaves and fruits when available)	Interviews with zoo zookeepers
			Charcoal (from burned twigs/trees) Water consumption from channel		Interviews with zoo zookeepers
Contacts with humans, other NHPs species and prey	Cameroon	*Physical contact and close proximity*	With humans: Hunting/Injuries/Pets, likely low influence on great ape intestinal microbiome Potential conflicts between gorillas and chimpanzees (rare)		^47^ Semi-structured interviews with local populations
			Potential hunting behavior (of monkeys, duikers, rodents and pangolins)	No mammal hunting reported	^48^ (based on observations at Lope National Park, Gabon and Nouabale-Ndoki National Park, Republic of Congo)
		*Environmental contact and spatial overlap*	Spatial overlap with humans and 7 other NHP species: *Cercopitecus nictitans, Cercopithecus cephus, Cercopithecus sclateri, Cercopithecus neglectus, Cercocebus agilis, Colobus guereza, Lophocebus albigena* Feeding on the same trees or in the same areas between gorillas and chimpanzees when wild mangoes are highly available		^47,49–51^ Semi-structures interviews with local people
	Zoo	*Physical contact and close proximity*	Veterinary care Daily close proximity between zookeepers and great apes (separated by fences/cages) Medical Training		Interviews with zoo zookeepers and direct observations
			Frequent physical contact (almost daily) with zookeepers through the grid (mutual grooming and play)	No physical contact	
		*Environmental contact and spatial overlap*	Cleaning procedures: Every 3 days Hot water at high pressure New straw No bleach	Island shared with *Colobus guerez* and *Cercopithecus ascanius* Cleaning procedures: Every 5 days Scrubbed with water and soap New straw No bleach	Interviews with zoo zookeepers and direct observations
			High probability of human contamination with fomites through foods, enrichment and structures Water channel shared by 32 other NHP species of zoo		Interviews with zoo zookeepers and direct observations

#### Living conditions

The published literature on which we relied shows that sympatric gorillas and chimpanzees in Central Africa tend to occupy different habitats: chimpanzees primarily inhabit mixed mature forests with closed canopies, whereas gorillas live in open canopy forests with dense herbaceous vegetation^36–39^. In our study region, however, our Cameroonian informants living in close proximity to great apes added that both species ranged near villages, in mosaics of cultivated land and young forest regrowth. Multiple informants explained that gorillas often avoid zones inhabited by chimpanzees out of fear: hunters claimed that chimpanzees, although smaller, could kill gorillas.

At the zoo site, the housing conditions of chimpanzees and gorillas are similar. Both species inhabit a species-dedicated indoor enclosure situated on an island, providing gorillas and chimpanzees with natural vegetation surrounded by a closed water channel. This water is not filtered or treated, nor is the channel cleaned, although gorillas and chimpanzees have access to piped, filtered water in their indoor enclosures. Indoor cleaning with water does occur, notably with hot water at high pressure and new straw every three days for chimpanzees; for gorillas, cages are scrubbed with water and soap and restocked with new straw every five days. The zoo does not use bleach to disinfect indoor areas of chimpanzees or gorillas. Zookeepers indicated that not using bleach resulted from their conviction that it would “allow their [great apes’] immune defenses [to] do the work, to stimulate their immunities.” The water used for enclosure cleaning is evacuated by pipes, not by the water channel. Outdoor enclosures are not cleaned, although zookeepers occasionally clear underbrush.

#### Diet

The different forest habitats occupied by chimpanzees and gorillas are partly due to their distinct feeding ecologies. Studies of Cameroon’s lowland gorillas show that they consume between 150 and 180 plant species, eating leaves, shoots, stems, pith, roots and bark from approximately 84 of these species; they also regularly eat invertebrates^40,52^. They may consume certain abundant staples year-round, as well as seasonal fruits when available, and foods of low nutritional value during the low fruiting season. Although dietary diversity may decline during the low fruiting season, which in some locations overlaps with the dry season but not others, this may not be the case in our study site. Our Cameroonian informants observed that gorillas pillaged forest gardens, particularly those with maize and cacao, during specific seasons. One farmer noted,

There are two seasons when (gorillas) pillage. In December, January, and February, when the trees flower but there is nothing to eat. If you cultivate maize then, they will go to eat it. And in cacao gardens (in November–December), when the fruits turn red, they will suck on the cacao “seeds”. They go back and forth between the forest and the gardens.

The forest chimpanzee diet is equally diverse, although chimpanzees tend to eat more fruit than gorillas^40–42^. South of our study site, in Lopé (Gabon) chimpanzees consumed 132 plant species, as well as invertebrates, mammals and honey^43^. In contiguous sites, chimpanzees consume 50 to 60 percent of the same fruits as sympatric gorillas^40,43,53,54^. Our Cameroon interviews indicated that chimpanzees also pillaged forest gardens, but were more selective in their choices. Whereas one interviewee observed, “when gorillas enter a field, it’s a massacre,”, another argued that “Gorillas don’t make choices. They will just wreck everything,” and that “chimps will select ripe bananas, climb up the trees, peel and eat them.” Unlike for gorillas, however, they did not point to a seasonality of chimpanzee pillaging.

According to zoo feeding lists and interviews with zookeepers, zoo chimpanzee and gorilla diets are similar, consisting of approximately 80% vegetable matter and 20% fruit, including 15 to 20 food species or preparations, although gorillas receive more fiber than chimpanzees. Both species also receive “Old World monkey” chow daily. Chimpanzees receive weekly animal protein (boiled eggs, cooked poultry), and occasionally seeds (wheat, maize, sunflower, almond, peanuts, walnuts, oatmeal), flour worms, boiled potatoes or bread. Gorillas consume daily a locally-made biscuit and a supplemental provision of twigs, leaves, and bark. They receive a weekly ration of nuts (e.g. almonds) and seeds, and in winter, a supplement of wheat flour and honey. Gorillas and chimpanzees also consume respectively four and six plant species growing on their islands, taking in flowers, fruits, leaves, bark, and herbs. Both occasionally drink the same water flowing through the surrounding channels, but more frequently piped water in their enclosures.

Although quantitative comparison of different diet compounds is not feasible, our qualitative diet analysis suggests a higher similarity between zoo chimpanzee and gorilla diets than those in the Cameroonian forest, and higher dietary diversity in Cameroon than in the zoo.

#### Contacts with humans, other NHPs species and prey

In southeastern Cameroon, chimpanzees and gorillas are not habituated to human beings, but they do share the forest with human inhabitants, who rarely hunt them and very rarely take juveniles as pets^47^. According to our observations and published research, spatial overlap is important, but physical contact between living great apes and humans is rare^47^. Hunting usually results in the death of the great ape.

Gorillas and chimpanzees have contact with one another, with other NHP species, and with potential prey. According to local informants, direct encounters between chimpanzees and gorillas may occur near fruiting trees and result in physical violence, an observation confirmed in published literature^49–51^. According to one group interview, “The chimp is really nasty. We once found the body of a gorilla out at N—(place name), killed by a chimp. Chimps always win battles with gorillas…They take up sticks and hit gorillas, or they throw rocks at them.” Chimpanzees and gorillas also share overlapping habitats with seven other NHP species: *Cercopitecus nictitans, Cercopithecus cephus, Cercopithecus sclateri, Cercopithecus neglectus, Cercocebus agilis, Colobus guereza, Lophocebus albigena*^47^.

In the zoo, chimpanzees and gorillas live separately and never share zoo spaces, although their habitats are ecologically similar. Gorillas and chimpanzees have access to the same water flow, which circulates through channels around their respective islands and is shared by the park’s 32 other NHP species. Although chimpanzees do not share their island with other primate species, gorillas cohabit with *Colobus guereza* and *Cercopithecus ascanius*, species that also inhabit the Cameroon forests.

Contact between humans and great apes in the zoo varies, occurring primarily through human preparation of animal feeding rations. Physical contact rarely occurs between gorillas and humans, except during medical interventions. In contrast, we observed daily contact between chimpanzees and zookeepers through mutual grooming and play.

### Microbiota diversity and composition

#### Microbial richness and diversity (alpha diversity) is related to environment

**Figure 1:**
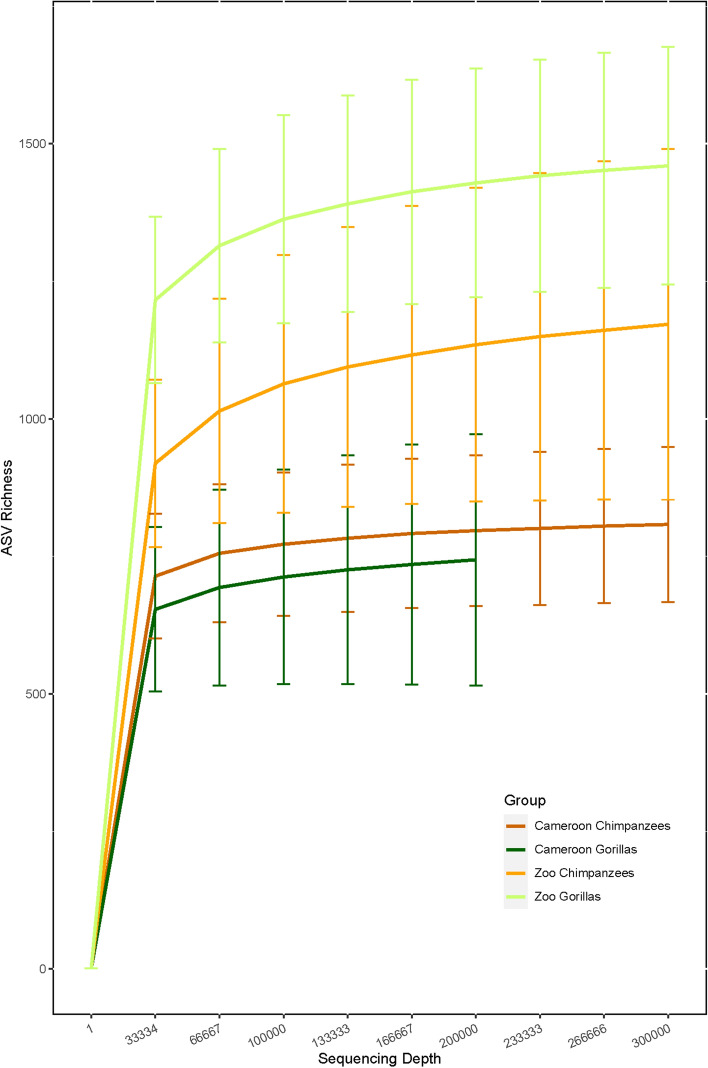
Mean rarefaction curves for each group (species/site). Error bars represent standard deviation.

**Figure 2:**
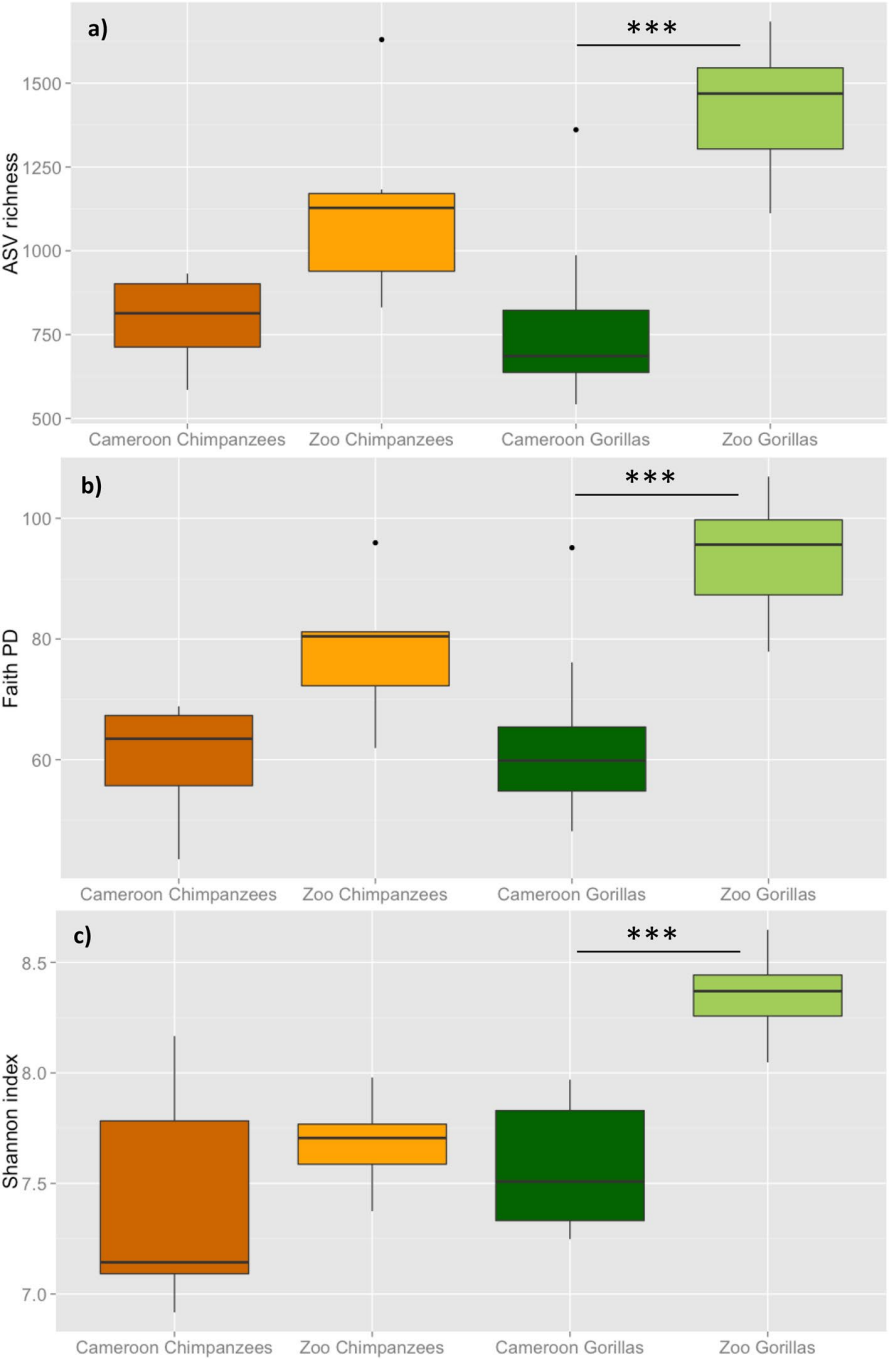
Boxplots of mean alpha diversity among Cameroon and zoo chimpanzees and gorillas for (**a**) ASV richness, (**b**) Faith PD index and (**c**) Shannon index. Pairwise comparisons were performed with Mann–Whitney U test and Bonferroni correction for multiple testing. P < 0.001 (***).

Microbial richness and diversity (observed ASVs, Faith PD and Shannon index) was significantly higher among zoo gorillas compared to Cameroonian gorillas (Mann–Whitney test, p < 0.001) but no significant differences were observed for chimpanzees (Figs. 1 and 2). The one chimpanzee who had received antibiotic treatment two months prior to sampling displayed an alpha diversity close to the mean (Supplementary Table S1).

#### Convergence of gut microbial composition among zoo chimpanzees and gorillas

**Figure 3:**
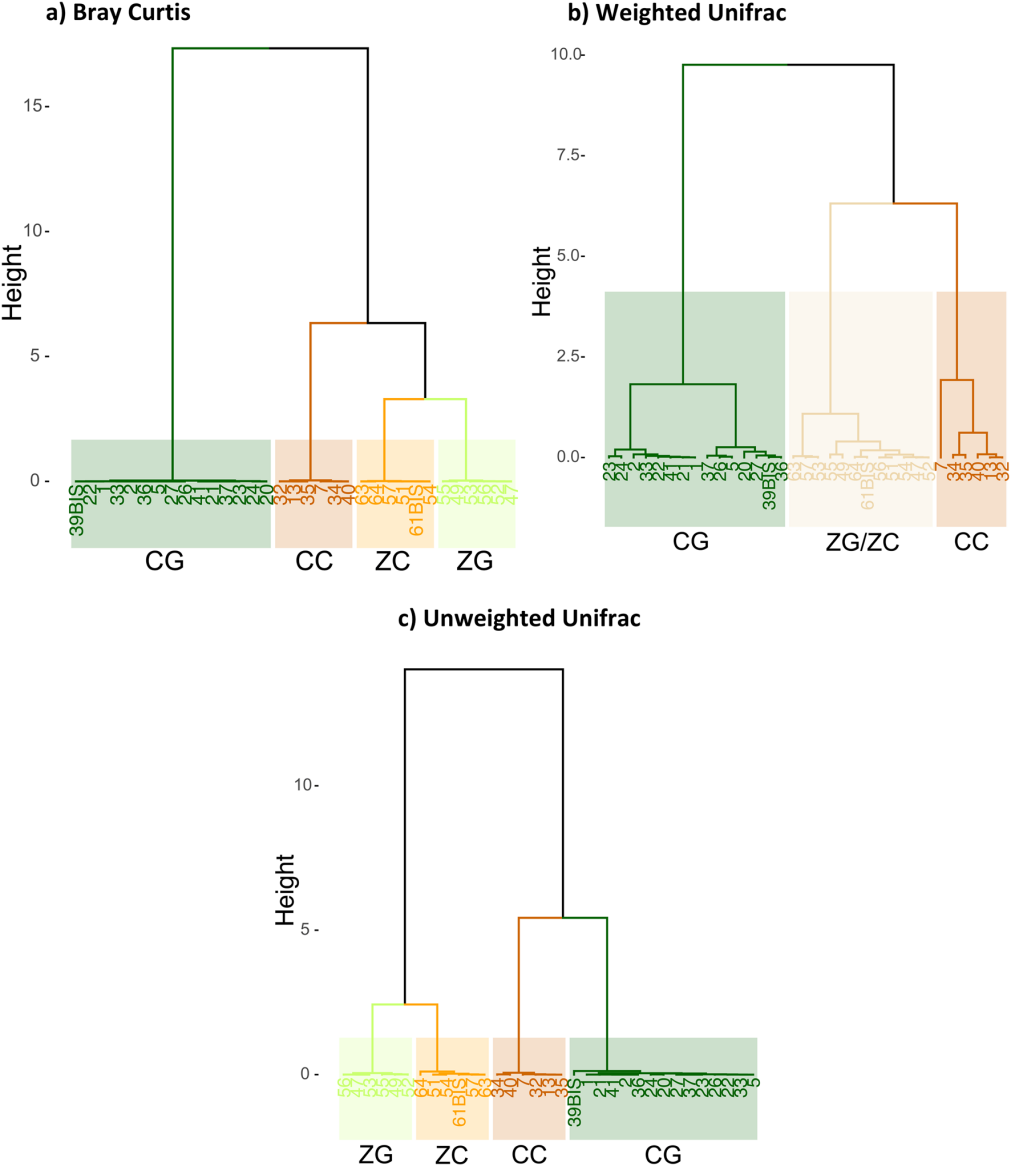
Dendrogram of Principal Component Analysis between samples based on: (**a**) Bray Curtis distance, (**b**) Weighted Unifrac distance and (**c**) Unweighted Unifrac distance. CG, Cameroon gorillas; CC, Cameroon chimpanzees; ZG, zoo gorillas; ZC, zoo chimpanzees.

Comparisons of gut microbial community composition using a PERMANOVA with Bray Curtis dissimilarity indices showed that the gut microbiota differed significantly across sites and species (all corrected p values < 0.005; Supplementary Table S2). Microbiota of zoo chimpanzees and zoo gorillas revealed the strongest similarity compared to the other two groups. Patterns were similar using both weighted and unweighted Unifrac indices (Fig. 3, Supplementary Table S2).

#### Taxonomic composition

On average, 100.0% of the analyzed 16S sequences were identified as bacteria or archaea classified at the phylum level, 94.6% at the family level and 72.3% at the genus level (Supplementary Table S3).

Firmicutes and Bacteroidetes phyla accounted for an average relative abundance of 78.8% (SD = 5.1; min = 59.7; max = 85.8) (See detailed phyla relative abundance for each group in Supplementary Figure S1).

**Figure 4:**
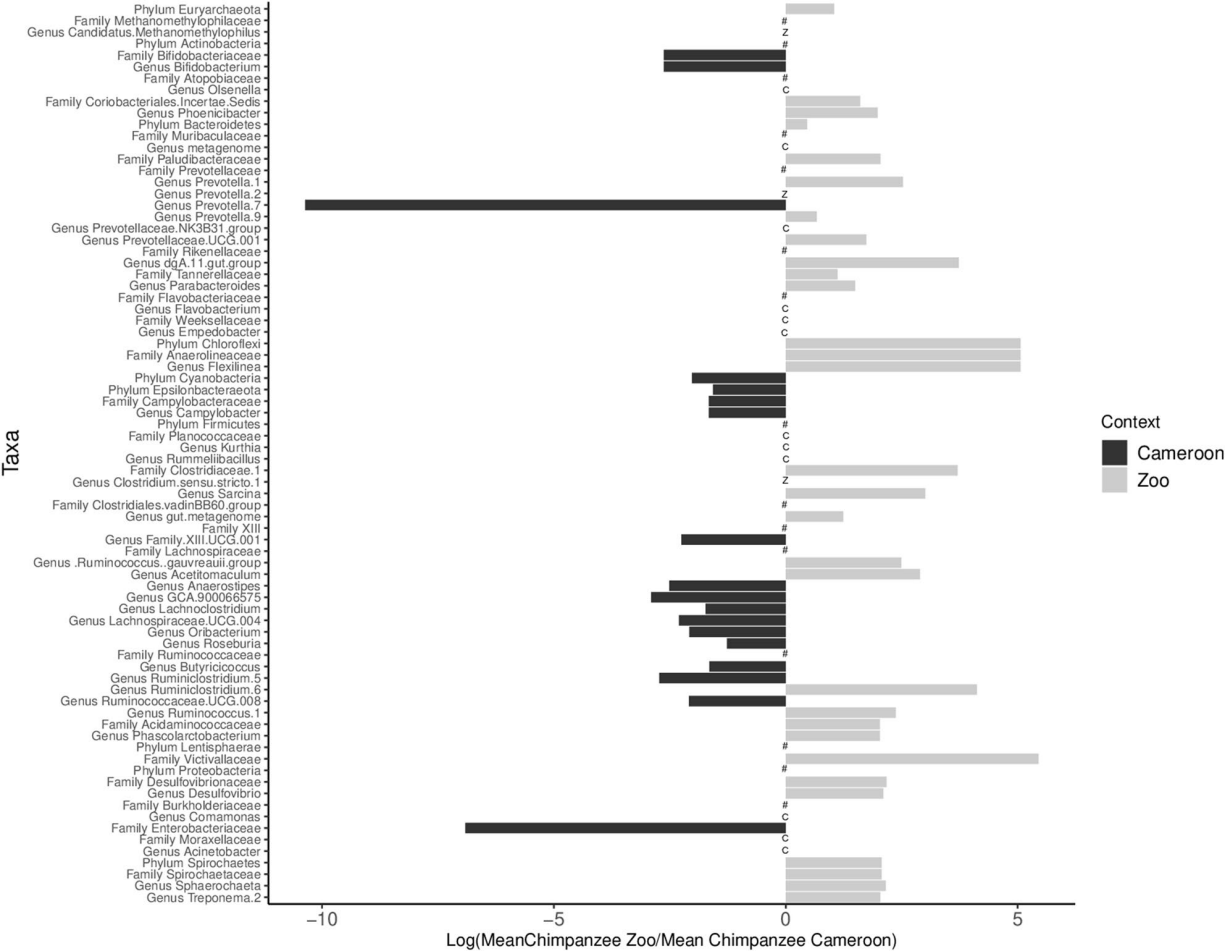
Phyla, families and genera with significant differences in relative abundance (corrected p-value < 0.05) between zoo (grey) and Cameroon (black) chimpanzees. The horizontal bars represent the ratio transformed in log10. C indicates the presence of taxa found only in Cameroon (absent in the zoo); Z indicates the presence of taxa found only in the zoo (absent in Cameroon); the # indicates no significant difference and the shows only phylum and family of corresponding genera.

**Figure 5:**
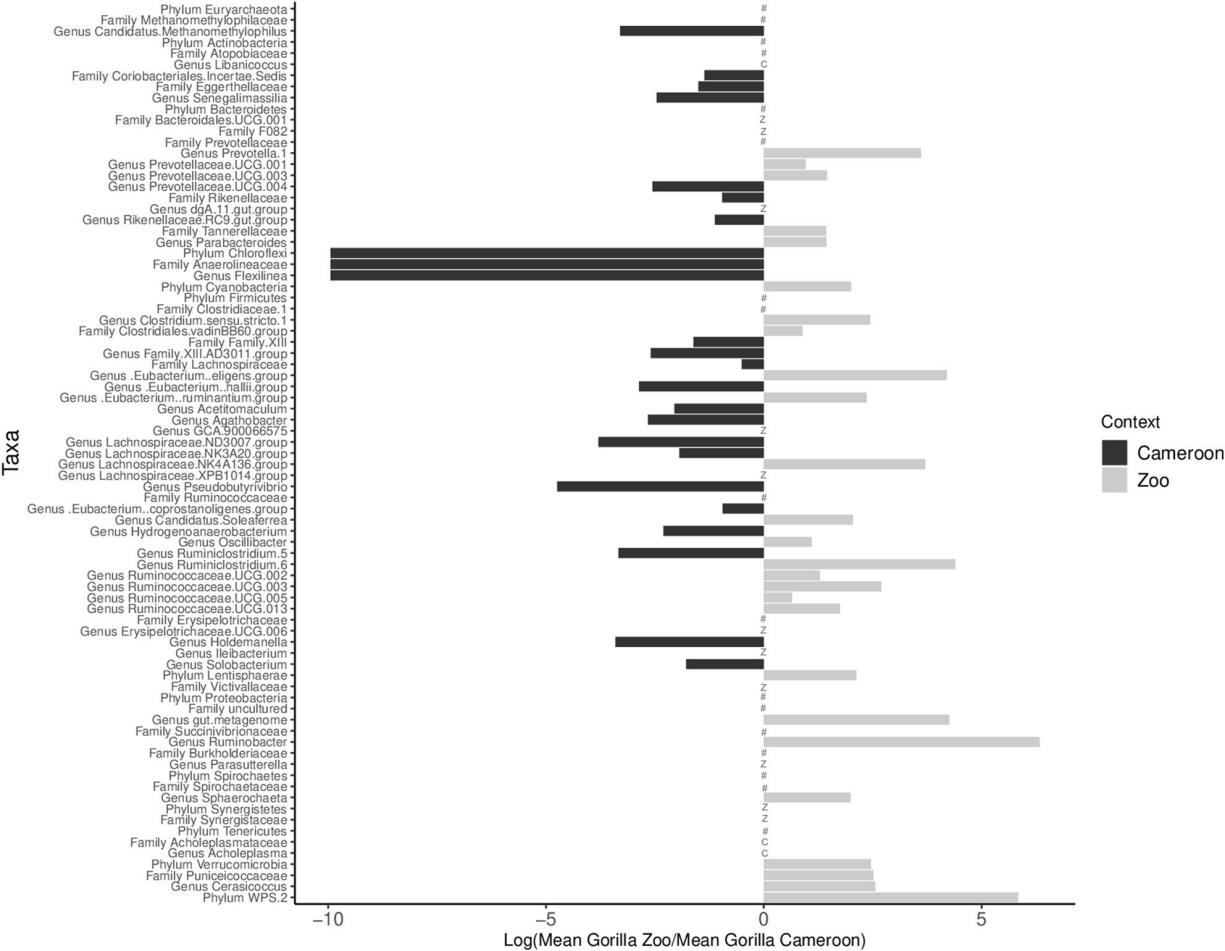
Phyla, families and genera with significant differences in relative abundance (corrected p-value < 0.05) between zoo (grey) and Cameroon (black) gorillas. The horizontal bars represent the ratio transformed in log10. C indicates the presence of taxa found only in Cameroon (absent in the zoo); Z indicates the presence of taxa found only in the zoo (absent in Cameroon); the # indicates no significant difference and the shows only phylum and family of corresponding genera.

Using Mann Whitney U tests, we investigated whether chimpanzees and gorillas had specific differences in the relative abundance of phyla, family and genera between zoo and forest settings (only for taxa with a mean relative abundance > 0.05%). Six phyla, 15 families and 42 genera in chimpanzees, and six phyla, 15 families and 44 genera in gorillas presented significant differences in relative abundance (Figs. 4 and 5, Supplementary Tables S4, S5 and S6). For instance, the phylum Chloroflexi had a 158-fold higher mean relative abundance in zoo chimpanzees compared to Cameroonian chimpanzees, represented by the Anaerolineaceae family and the *Flexilinea* genus. The most marked difference was observed for the Victivallaceae family, with a mean relative abundance 233 times higher in zoo chimpanzees compared to Cameroonian chimpanzees. The latter had much higher relative abundance of the *Prevotella 7* genus (almost 32,000 times higher) and *Enterobacteriaceae* family (1,000 times higher) compared to zoo chimpanzees.

Among gorillas, mean relative abundance of the phylum Chloroflexi was 21,000-fold higher in Cameroon than among those in the zoo, represented by the Anaerolineaceae family and the *Flexilinea* genus. Several genera from the phylum Firmicutes (Family Lachnospiraceae) were also more abundant in Cameroonian gorillas compared to zoo gorillas, including *Pseudobutyviribrio*, *Eubacterium halii* and *Agathobacter*. However, several genera from the same family were more abundant among zoo gorillas, including *Eubacterium ruminantium* and the Lachnospiraceae XPB1014 group.

Comparison of the two settings including both chimpanzees and gorillas showed higher rates of Euryarchaeota, Bacteroidetes, Lentisphaerae, Patescibacteria, Spirochaetes, and Verrucomicrobia in the zoo and higher rates of Actinobacteria, Epsilonbacteraeota, Firmicutes, and Fusobacteria in Cameroon (Supplementary Table S4).

## Discussion

Our study compares gut microbial diversity and composition of two sympatric great ape species in forest and zoo settings, employing an anthropological approach to hypothesize about their differences. Although we detected differences in gut microbial community composition across species and sites, contrary to our hypothesis, we did not observe lower gut microbial richness and diversity for either gorillas or chimpanzees in the zoo. In comparison with great apes in Cameroon, zoo gorillas demonstrated significantly higher gut microbial richness, whereas chimpanzees had similar gut microbial richness and diversity in both locations.

This finding contrasts with other studies, which find that “captivity” is associated with a loss of bacterial diversity and a shift in the gut bacteriome composition, possibly because of decreased dietary diversity and reduced contact with bacteria in water, soil, other animal species and dietary sources^2,24,27^. Nevertheless, three studies have conversely found *higher* alpha diversity among zoo animals. Campbell and colleagues concluded that gorillas and chimpanzees housed at an American zoo displayed higher gut microbial richness compared to those living in a Congolese forest^55^. For other animal taxa, McKenzie and colleagues observed higher gut microbial alpha diversity among zoo-housed Rhinocerotidae family (*Ceratotherium simum* and *Diceros bicomis*) and hypothesized specific host biological traits to explain this result; they did not evaluate specific environmental conditions under which these animals were sampled^27^. A third study found that leopard seals (*Hydrurga leptonyx*) had higher gut bacteriome richness under zoo conditions than in less-controlled settings, suggesting that environmental factors (especially diet) may enhance gut species composition richness^56^. However, none of these studies characterize conditions of captivity, despite suggestions in the literature that these conditions are likely to affect the gut bacteriome^33^.

Our anthropological data suggest that the specific zoo setting in which we sampled the apes may explain some of the patterns in our data. In our zoo site, we observed that great apes had substantial access to outdoor, vegetated areas, local soils, and a water channel shared by 32 other NHP species. Additionally, our interviews and observations of zookeepers revealed cleaning practices that contrast with those of many other zoos, in that they do not use disinfectant chemicals. As a result, it appears that our sampled zoo gorillas and chimpanzees may not have reduced contact with environmental microbial pools, as is often assumed for animals living under these confined conditions. Furthermore, the shared water channel may provide a means of increased microbial transmission between NHP species, even among those housed in different enclosures. Sharing a habitat—even indirectly—with 32 other NHP species, is unusual, and does not occur in unconfined conditions. Such insights could help to explain patterns of increased microbial richness. Moreover, although chimpanzees used the water channel and had closer, more frequent physical contact with zookeepers than did gorillas, zoo chimpanzees did not display higher alpha diversity. Interspecies contact cannot fully explain the differences observed.

Observed patterns in microbial richness may also result from differences in diet composition across sites, either generally or seasonally. We collected stool samples in Cameroon during the dry season, when diets more likely consist of lower species diversity. Reduced dietary diversity has been linked to reduced microbial diversity in unconfined primates^24^. Hence, if gorilla dietary diversity in the zoo exceeded that of Cameroonian gorillas during the dry season, that may explain our results. This mechanism has been suggested in another study of zoo great apes reporting similar patterns^55^. Nevertheless, studies of gorillas have come to different conclusions about the influence of seasonality on gut microbiome diversity^19,57^ and comparative studies of NHPs suggest that the influence of seasonality on the gut bacteriome among unconfined primates is less than that of host phylogeny. Although dietary differences among these diverse environmental conditions may contribute to the observed patterns, additional research is necessary.

Finally, a possible explanation for higher microbial richness in zoo gorillas is that those living in Cameroon may have suffered from gut dysbiosis at the time of sampling; this dysbiosis can result from adverse environmental, dietary, pathogenic, or other conditions^58–60^. We cannot confirm or set aside this suggestion, although no collected stool sample showed signs of diarrhea. Moreover, we found no predominance of unexpected bacterial taxa; dominant taxa detected were consistent with previous studies^19,61,62^.

With regard to overall gut bacteriome composition, our data indicate that each species in each site has a distinct bacteriome. This result convenes to those of other studies, which highlight the importance of phylogeny as a driver of gut microbial composition^23,25,33^. Nevertheless, we unexpectedly found that the gut bacteriome of zoo gorillas was more similar to that of zoo chimpanzees than that of Cameroonian gorillas. Similar to reported patterns of microbial diversity, our finding here may result from living conditions, diet, and/or inter-species contacts. First, our observations and interviews at the zoo revealed gorillas and chimpanzees had more similar diets than their counterparts in Cameroon, which could lead to a convergence in gut bacteriome^33^. Both published literature and our interviews indicated that Cameroonian gorillas consume much more vegetable matter than do chimpanzees, especially during the dry season when we collected our samples^42,43,63^. Cameroon gorillas appear to eat more leafy greens than their zoo counterparts, and *P. t. verus* in the zoo seem to consume more leafy greens than *P. t. troglodytes* in Cameroon. Our Cameroonian informants observed flexibility in great ape feeding sites and underscored different pillaging patterns, as well as seasonal gorilla predations in forest gardens. At the zoo, however, gorillas and chimpanzees eat largely the same diet, approximately 80% vegetable matter and 20% fruit.

Additionally, our anthropological data indicated that zoo gorillas and chimpanzees occupied nearly identical indoor and outdoor habitats, making it likely that these apes are exposed to similar environmental microbial communities. In contrast, in Cameroon, field interviews indicated that gorillas and chimpanzees tended to inhabit and use different forest types, although identifying sites frequented by chimpanzee groups, informants also noted important variability in habitat types, with some groups living near human settlements.

Finally, constrained social structures and physical or indirect contact with other host species in zoo conditions may also affect intergenerational transmission within a given host species that dampens species-specific patterns in gut bacteriome composition that we would have expected.

Interviews and participant-observations of zookeeper and Cameroonian interactions with gorillas and chimpanzees provided crucial evidence of direct and indirect contact with humans and other NHPs. Zoo great ape contact through the water channel with numerous NHP species may also affect microbial diversity and dampen some host species-specific patterns that might be observed in unconfined environments. This question requires further study.

Beyond the convergence of gut bacteriomes among zoo chimpanzees and gorillas, the differences we observed in the gut bacteriome of each great ape group provide insight into the relationship between host ecology and potential bacteriome function. For example although both zoo and Cameroonian gorilla guts contained abundant Lachnospiraceae and Ruminococcaceae families, which play an important role in the fermentation of dietary fibers^64^, the most abundant genera from these families differed according to site. This result likely reflects the distinct dietary vegetable matter and fiber in the two settings. Similarly, patterns in the *Chloroflexi* phylum (represented by the genus *Flexilinea*, previously identified in the SHD 231 group^65^) were likely associated with dietary differences. *Flexilinea* was much less abundant in zoo gorillas than in Cameroonian gorillas; that pattern was reversed in chimpanzees, with higher relative abundance among zoo chimpanzees. Differences in this taxon between gorillas and chimpanzees, and within gorillas across seasons, have been previously associated with diet^19,55^. Although we cannot link its relative abundance to a specific ape dietary component in this study, our interviews and the literature suggest that zoo gorillas had reduced leafy material in their diets compared to those in Cameroon, whereas zoo chimpanzees had more leafy material in their diets compared to Cameroonian chimpanzees. Finally, although we did not find higher relative abundance of *Bacteroides* among zoo great apes compared to Cameroonian ones^2^, they did have higher relative abundance of several *Prevotella* strains. *Prevotella* is also known to shift in response to diet, particularly dietary carbohydrates such as starch, which is more likely to comprise a higher proportion of zoo diets in the form of “Old World Monkey” chow.

Our study has several limitations. First, the chimpanzees at each site were not the same subspecies (*P. t. verus* at the zoo, *P. t. troglodytes* in the Cameroonian forest). This difference could have affected our results, given the effect of host phylogeny on the primate gut bacteriome^66^. Any effect on our results from this phylogenetic difference, however, are likely to be subtle because these host differences occur at the sub-species level. Moeller and colleagues have shown that the differences between two chimpanzee subspecies (in unconfined settings) are much lower compared to differences between two species or two genera of great apes^61^. Additionally, host phylogeny has not been shown to strongly influence microbial diversity within primate families or genera^66^. Second, we did not collect highly detailed data on alimentary regimes of gorillas and chimpanzees in Cameroon, primarily because this study was part of a much broader one that did not undertake an in-depth investigation of primate dietary ecologies. Nonetheless, we report habitat and dietary data collected through anthropological observation and field interviews and put these results into dialogue with our gut microbial analyses. Third, our sample sizes from the forest and zoo are not large, because of labor and time limitations in Cameroon and because of limited chimpanzee and gorilla population sizes in our zoo site. Nevertheless, our samples came from relatively cohesive groups at each site, which allowed us to document carefully their environmental conditions. Fourth, we did not perform negative controls. We cannot rule out contamination, but all samples were collected according to the same method, and all samples were processed for extraction, NGS libraries and sequencing according to the same protocol. Finally, we did not conduct meta-analyses with other published sequences because these sequences do not also include meta-data on the conditions within which NHPs were living.

Prior comparative studies of nonhuman primate gut microbiome have focused much attention on comparing diversity and composition, linking explanations of difference to categories of “wild”, “captive”, “pristine”, “disturbed”^2,26,28^. Categories of “wild” and “captive” are not homogeneous or stable across all settings. Nor are they sufficient to capture the substantial diversity of conditions under which great apes and other NHPs live. To address this gap, one unusual dimension of our analysis was the use of anthropological methods and integration of Cameroonian and zookeeper experience and knowledge into our study. Our careful evaluation of sampled populations employed targeted interviews and participant-observations in Cameroon and the zoo to complement our quantitative data. In particular, based on their long experience living in proximity with great apes, Cameroonians provided nuanced description of ape habitats, pillaging patterns, and gorilla-chimpanzee interactions that have not appeared in published literature. This approach helped to push our comparative bacteriome analyses beyond description of results and to suggest specific environmental, dietary, and inter-species contact conditions that may explain the results, and should certainly constitute the focus of further investigation. Stool collection in Cameroon could not have been performed without Cameroonians, who know the forest intimately and could guide us to sites frequented by gorillas and chimpanzees.

Our analysis and reflections lead to multiple recommendations for future investigations. Studies evaluating the influence of environment on animal microbiomes should collect detailed data that tease out the influences of specific living conditions, alimentary practices and inter-species contacts on gut microbiome. Studies should also include systematic environmental sampling of habitats. This combination of multiple sample types, complemented by other disciplinary approaches, will substantially improve our ability to draw robust conclusions and permit insight across multiple settings. Anthropological methods can document critical processes and practices^67^, and ecological tools can reveal crucial living conditions and dietary and inter-species factors, all of which allow us to move beyond categories about host habitats or diet that obscure more than they reveal.

## Conclusions

Our comparative analysis of the gut bacteriome among gorillas and chimpanzees in a Cameroon forest and a European zoo showed significantly higher microbial richness and diversity among zoo gorillas, in comparison to Cameroonian forest gorillas, but no difference for chimpanzees. Although phylogeny is a strong driver of species-specific microbial composition, we suggest that environmental conditions may contribute to our results, especially to gut microbial similarity of zoo gorillas and chimpanzees. More generally, we argue that categories “wild” and “captive” illuminate less about the conditions under which mammals are sampled than is frequently assumed. Social sciences tools that can describe carefully the diverse environmental conditions under which samples are collected may facilitate better understanding of divergent results of mammalian gut bacteriome comparisons.

## Material and methods

### Study sites and sampling methods

**Figure 6:**
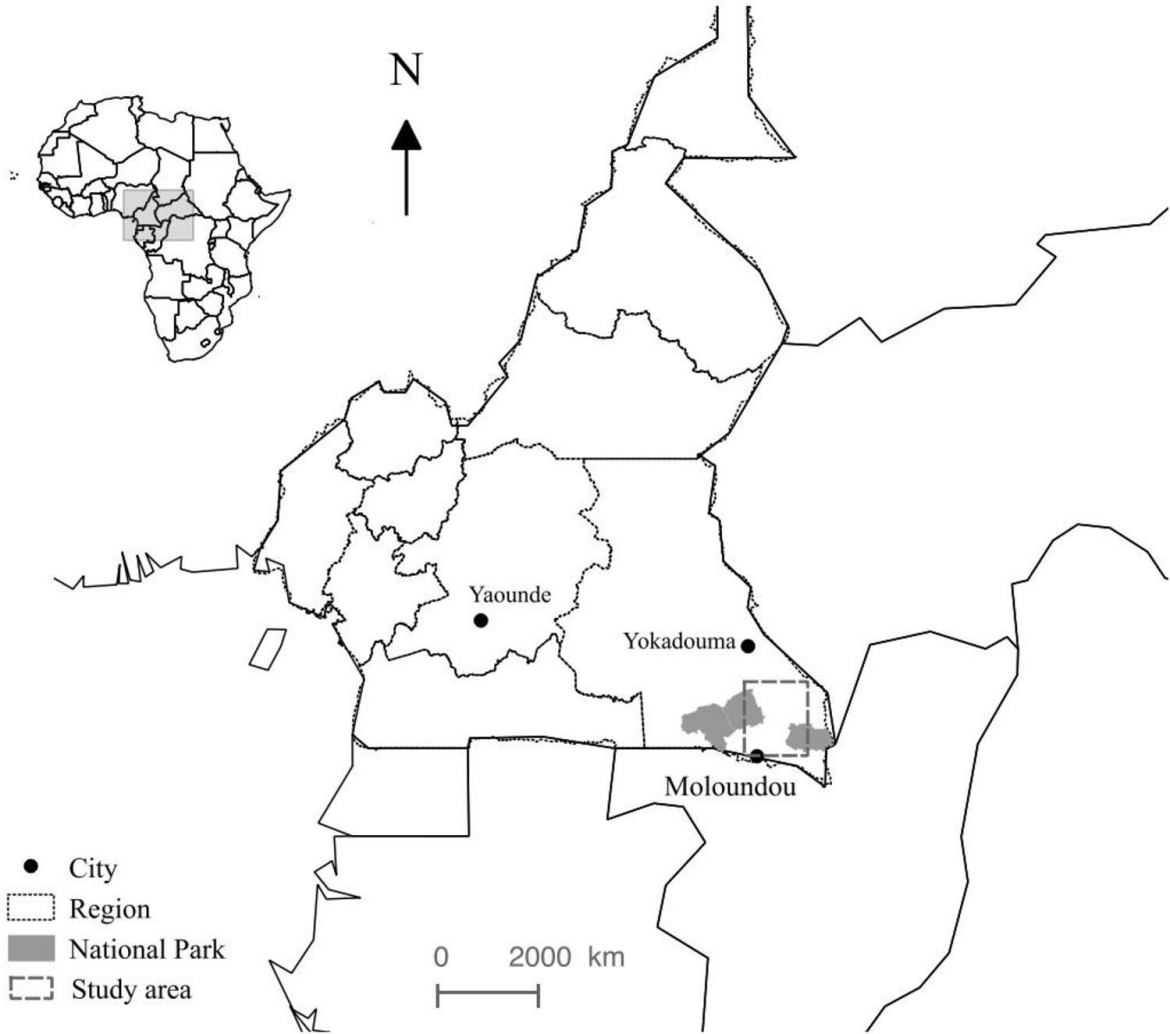
Location of the study area, southeastern Cameroon. The map was developed with QGIS software v. 3.4.3 (https://qgis.org/fr/site/). Sources of layers: www.wri.org (cities and regions); www.protectedplanet.net (protected areas).

To protect the anonymity of human populations and enterprises associated with these sites, we do not report the specific names of the forest and zoo sites. The forest site in southeastern Cameroon is located in a zone between the towns of Yokadouma and Moloundou (Fig. 6) where gorillas (*Gorilla gorilla gorilla*) and chimpanzees (*Pan troglodytes troglodytes*) live sympatrically^68^. With local trackers, VN collected fecal samples in the dry season of January 2016. In the daytime, the field team identified fresh traces of gorillas and chimpanzees, paying particular attention to these species’ vocalizations. The team set up a night camp close (within approximately 250 m) to targeted species’ group nesting site. In the early morning, the team pinpointed more precisely the group’s location and collected fresh fecal samples from the nighttime nests. All stools were collected fresh, less than 12 h from emission. Parts of stools with any ground contact were not collected. To avoid multiple sampling of individuals, only a single, fresh dung sample was extracted from each nest.

The European zoo site houses both gorillas (*G. g. gorilla*) and chimpanzees (*Pan troglodytes verus*, a different sub-species from that located in southeastern Cameroon). In November 2017, working with zookeepers in the early morning hours, VN collected fecal samples from zoo chimpanzees and gorillas. Only feces emitted in the presence of zookeepers were collected, to identify its originator and to avoid multiple sampling of the same individual(s).

All gorilla and chimpanzee feces collected in the forest and zoo had a normal consistency; no signs of diarrhea, blood, mucus or macro-parasites were detected. One adult male, zoo-housed chimpanzee had received antibiotic treatment (amoxicillin/clavulanic acid) for a respiratory infection two months prior to fecal sampling (2017-B-01).

After collection, forest and zoo stool samples were immediately stored in RNA later tubes at room temperature^69^ and frozen at − 80 °C within 10 days, the Cameroonian samples at the Centre Pasteur du Cameroon in Yaounde, and the zoo samples at the Hôpital Saint Louis in Paris. To conceal the species and collection site, each sample received a code. Supplementary Table S1 details the metadata of fecal sample collection.

### DNA extraction, amplification and sequencing

The laboratory received all frozen samples with codes and was blinded about species origin. RNA later tubes containing fecal samples were centrifuged for 30 min at 4400×*g* to separate the different phases (solid, organic and aqueous). DNA was extracted from 0.25 g of the stool sample with the QIAamp Powerfecal DNAextraction kit (Qiagen, Hilden, Germany), following manufacturer instructions. Amplification of region V3-V4 of the 16S rRNA gene and library preparation was performed, as described in Illumina protocol for 16S sequencing with primers S-D-Bact-0341-b-S-17 and S-D-Bact-0785-a-A-21^70^. Libraries were sequenced on the Illumina MiSeq platform (Illumina, San Diego, California) with a MiSeq Reagent kit V3.

### Sequence analysis

Sequencing yielded 35,215,908 raw sequence reads (average of 1,035,762 per sample, range of 403,841–3,591,434). Low quality reads were trimmed using Trimomatic v.0.35 59 with an average quality threshold set to 25. Trimmed reads were merged using Casper v0.8 60. Merged reads were processed in QIIME2 2020.6^71^. Sequences were trimmed, quality-filtered, and dereplicated using the DADA2 algorithm^72^. Sequences were truncated at 240 base pairs, and a maximum of four errors were permitted for both forward and reverse reads. DADA2 was simultaneously used to merge paired reads and infer amplicon sequence variants (ASVs). Taxonomy was assigned in QIIME2 using a Naive Bayes classifier trained on the SILVA 132 database using full 16S rRNA gene sequence lengths^73^. Mitochondria and chloroplast ASVs were filtered from the dataset. After all filtering steps, our dataset contained 23,807,353reads with an average of 700,216 per sample (range 201,807–7,732,097). We generated alpha rarefaction curves using alpha-rarefaction, but given the sampling depth, we did not discard any samples and chose to rarefy our data at an even 200,000 reads per sample. We calculated microbial richness and Shannon and Faith phylogenetic diversity measures using the core-metrics-phylogenetic plug-in in QIIME2. The same command generated Bray Curtis and unweighted and weighted UniFrac distance matrices describing pairwise similarity between samples.

The Principal Component Analysis and cluster dendrogram using the Bray Curtis dissimilarity index were used to visualize the data in R studio^74^ with the package FactoMineR^75^. The cluster dendrogram was also performed on weighted and unweighted Unifrac indices. Permutational analysis of variance (PERMANOVA) was used to compare microbial communities between each group based on Bray Curtis dissimilarity indices, weighted and unweighted Unifrac distances using the adonis2 function in the vegan package^76^, as well as the PairwiseAdonis package^77^. Comparison of alpha diversity indices and relative abundances for each taxa level, between groups (species/site), were conducted with the Mann–Whitney U test, with Bonferroni correction for multiple testing.

### Environment, diet and interspecies contact

In our two study sites, we employed qualitative data through anthropological participant-observation and semi-directed interviews (with open-ended questions) on factors that could influence gorilla and chimpanzee gut microbiota: environment, diet and interspecies contact. Comparing these factors offered grounds for gaining insight into the significance of our microbiota analysis.

Habitat and dietary data for great apes in Cameroon were collected over multiple field visits, through semi-directed interviews (Supplementary Methods S1) with people living in proximity to these animals and an extensive published literature on great ape ecologies in the northern Congo basin forest. Interspecies contacts were investigated through quantitative data analyses, and semi-structured interviews were conducted in French or in the Bangando language; all interviews were recorded. Detailed notes were collected for participant-observations.

In the zoo setting, VN and TG-V spent five days observing and documenting living conditions among the great apes sampled, including feeding regimens, enclosed and open living conditions, and cleaning practices of these habitats. In the zoo, we conducted direct observation and semi-structured interviews with zookeepers to document great ape contacts with other animal species, including humans.

We conducted coding of all qualitative data to identify habitats, feeding practices, and human perceptions of ecologies and capacities.

### Ethical approvals

The Cameroon National Research Ethics Board for Human Health (Decision no. 2015/05/598/ CE/CNERSH/SP) and a Committee for the Protection of Persons (2017-A00734-49) reviewed the protocol and provided ethical approval for this study. We also received authorization to conduct the study from the Cameroon Ministry of Public Health. All participants, after receiving a written and oral description of the study and their rights, signed an informed consent form. All methods were carried out in accordance with relevant guidelines and regulations.

## Supplementary information

Supplementary Information.

## Data Availability

The data analysed in this manuscript are available on simple request without restriction. Recordings of semi-structured interviews are not available because our ethical approvals and consent did not allow for sharing of this data. Genetic sequences are deposited on Sequence Read Archive (Accession number PRJNA666756).
