# Supplementary material for: A multi-disciplinary comparison of great ape gut microbiota in a central African forest and European zoo
Source: Sci Rep. 2020 Nov 5;10:19107. doi: 10.1038/s41598-020-75847-3 (PMC7645722; doi:10.1038/s41598-020-75847-3)
Supplement: Supplementary file 1 — Supplementary Information. [file 41598_2020_75847_MOESM1_ESM.pdf]

Title : A multi-disciplinary comparison of great ape gut microbiota in a central African forest and European zoo

Victor Narat[1,2]*, Katherine R Amato[3,4]*, Noémie Ranger[5], Maud Salmona[5, 6], Séverine Mercier Delarue[5], Stephanie Rupp[7], Philippe Ambata[8], Richard Njouom[9], François Simon[5], Tamara Giles-Vernick[2, 4**], Jérôme LeGoff [5, 6**]

Corresponding authors: Jérome LeGoff and Tamara Giles-Vernick, tamara.giles-vernick@pasteur.fr

[1] Eco-anthropologie, UMR7206 CNRS/MNHN/Paris Diderot, Site du Musée de l'Homme, Paris, France.

[2] Institut Pasteur, Anthropology and Ecology of Disease Emergence Unit, Paris, France

[3] Department of Anthropology, Northwestern University

[4] Humans and the Microbiome, CIFAR, Toronto, Canada

[5] Université de Paris, Equipe INSIGHT, Inserm U976, F-75010 Paris, France

[6] Département des Agents Infectieux, Virologie et Greffes, AP-HP, Hôpital Saint-Louis, F-75010 Paris, France

[7] Department of Anthropology, City University of New York – Lehman College, New York, NY

[8] Ministry of Agriculture and Rural Development, Yaounde, Cameroon

[9] Centre Pasteur du Cameroun, Yaounde, Cameroon

* These authors contributed equally to this work

** These authors contributed equally to this work

Supplementary Table S1: QIIME2 sequence data processing summary by sample

Extraction ID	Sample ID	Site	Species	Input sequences	Quality filtered sequences	% of input passed filter	Denoised sequences	Pair end merged sequences	% of input merged	Non chimeric sequences	% of input non chimeric	Number of ASV after rarefaction at 200,000 reads
**7**	FB-002	Cameroon	Chimpanzee	779377	614206	78.81	612650	548023	70.32	492131	63.14	585
**13**	FB-004	Cameroon	Chimpanzee	1071327	828930	77.37	823161	688240	64.24	613704	57.28	686
**32**	FB-006	Cameroon	Chimpanzee	892730	781969	87.59	776915	706611	79.15	648157	72.6	834
**34**	FB-005	Cameroon	Chimpanzee	1004960	859909	85.57	853105	759498	75.57	701968	69.85	924
**35**	FB-001	Cameroon	Chimpanzee	977282	854903	87.48	848844	773779	79.18	687625	70.36	932
**40**	FB-003	Cameroon	Chimpanzee	883361	777470	88.01	772897	688985	78.00	619858	70.17	793
**51**	2017-B-05	Zoo	Chimpanzee	596845	470296	78.8	464659	412687	69.14	383835	64.31	878
**54**	2017-B-02	Zoo	Chimpanzee	1106236	945969	85.51	938455	804366	72.71	752581	68.03	1134
**57**	2017-B-01	Zoo	Chimpanzee	894835	765643	85.56	757274	638060	71.3	574249	64.17	1122
**63**	2017-B-03	Zoo	Chimpanzee	938472	824845	87.89	818624	714811	76.17	661436	70.48	1183
**64**	2017-B-06	Zoo	Chimpanzee	1081546	929799	85.97	924640	819432	75.76	735932	68.04	831
**61BIS**	2017-B-04	Zoo	Chimpanzee	3591434	3437099	95.7	3420867	3219109	89.63	2732097	76.07	1630
**1**	EA-001	Cameroon	Gorilla	887030	685547	77.29	681637	536280	60.46	498714	56.22	545
**2**	DA-007	Cameroon	Gorilla	689517	538812	78.14	536631	445122	64.56	436312	63.28	589
**5**	DA-009	Cameroon	Gorilla	858214	639931	74.57	634272	498936	58.14	457021	53.25	646
**20**	DA-003	Cameroon	Gorilla	764833	593154	77.55	589026	508728	66.51	495227	64.75	813
**21**	EA-004	Cameroon	Gorilla	838798	627393	74.8	623715	463292	55.23	429555	51.21	542
**22**	EA-002	Cameroon	Gorilla	1027815	783137	76.19	776270	569320	55.39	488506	47.53	629
**23**	EA-005	Cameroon	Gorilla	1107270	951438	85.93	945956	842316	76.07	791705	71.5	673
**24**	EA-003	Cameroon	Gorilla	991161	855999	86.36	850603	741905	74.85	677583	68.36	645
**26**	DA-004	Cameroon	Gorilla	921334	808300	87.73	802937	712469	77.33	667638	72.46	987
**27**	DA-001	Cameroon	Gorilla	1187800	1040254	87.58	1033460	922825	77.69	867548	73.04	923
**33**	EA-006	Cameroon	Gorilla	906695	773332	85.29	767822	667802	73.65	597806	65.93	686

**36**	DA-005	Cameroon	Gorilla	716923	590660	82.39	587010	541142	75.48	518576	72.33	724
**37**	DA-002	Cameroon	Gorilla	739696	594459	80.37	590371	527602	71.33	510247	68.98	832
**41**	DA-008	Cameroon	Gorilla	999521	874750	87.52	868974	768893	76.93	710259	71.06	704
**39BIS**	DA-006	Cameroon	Gorilla	2935809	2808803	95.67	2787968	2639966	89.92	2284488	77.81	1361
**47**	2017-C-05	Zoo	Gorilla	912566	777368	85.18	766861	629828	69.02	578951	63.44	1568
**49**	2017-C-02	Zoo	Gorilla	990595	871869	88.01	863567	729787	73.67	682077	68.86	1459
**52**	2017-C-04	Zoo	Gorilla	801034	627113	78.29	620344	524793	65.51	493683	61.63	1252
**53**	2017-C-06	Zoo	Gorilla	1098832	926930	84.36	917589	782543	71.22	744054	67.71	1684
**55**	2017-C-01	Zoo	Gorilla	742989	638399	85.92	632928	520665	70.08	480306	64.65	1112
**56**	2017-C-03	Zoo	Gorilla	875230	745493	85.18	736387	625597	71.48	591717	67.61	1479

Supplementary Methods S1: Semi-directed interview guides

For great ape zookeepers

1. How long have you worked at this zoo?
2. How long and in what capacity have you worked with this species of great ape?
3. Can you describe for me a typical day working with these animals?
4. Do your daily activities change seasonally, and if so, how?
5. Can you tell me more about feeding of these animals? (timing, dietary composition and quantities for each animal by age and sex, seasonal changes, other relevant practices)
6. From where do you obtain these foods?
7. How do you organize their time outdoors? Are there ever times when they do not go outdoors?
8. Do they eat plant material in the outdoor enclosure or drink from the canal around the island?
9. Do you provide additional supplements from time to time? What?
10. Can you describe any daily physical interactions that you have with these animals? Do you ever touch them? When and why?
11. Have these great apes been sick recently (within the last two months)? If so, which one(s)? What did you do? Did you provide treatment, and if so, what?
12. In your experience, is it possible for disease to pass between humans and captive animals in this zoo setting? Why or why not? Have you ever witnessed such transmission, and if so, when?

For inhabitants of southeastern Cameroon

1. What environmental changes have you observed or heard about in this forest ? Over what period of time ? What do you think of these changes ?

2. In your language, how do you refer to a gorilla/chimpanzee?
3. Where do gorillas/chimpanzees reside in this forest? What are their preferred habitats? Do they stay in the same place or do they move around?
4. Do you find gorillas/chimpanzees in close proximity to your village (use map)?
5. Do you hunt gorillas/chimpanzees? Were these animals hunted in the past? When, and by whom?
6. Who hunts gorillas/chimpanzees now?
7. What weapons are used to hunt gorillas/chimpanzees?
8. How are gorillas/chimpanzees usually butchered? Where and by whom?
9. What do you do with the different parts of the animals?
10. Can you describe for me the qualities/personality traits of gorillas/chimpanzees?
11. How do they behave with one another? Have you observed their behavior? What features strike you as most important?
12. Do gorillas/chimpanzees have certain kinds of powers? Which ones ? These powers, have they changed over time?
13. Do gorillas/chimpanzees have knowledge? Of what?
14. What do people think about these animals?
15. How do you behave when you meet one? Why?
16. How do gorillas/chimpanzees behave when they see you?
17. Do people and gorillas/chimpanzees share certain capacities or knowledge ?
18. Do you think that relations between people and gorillas/chimpanzees have changed ? If so, since when ? and why ?
19. Do gorillas/chimpanzees ever fall sick ? How do you know?

20. What do gorillas/chimpanzees do when they are sick ? How do they take care of themselves? (If animal uses leaves for self-treatment, ask person to show leaves. Ask if people use these leaves as well.

21. Is it possible for an illness to be transmitted from a gorilla/chimpanzee to a human? Or from a human to a gorilla/chimpanzee? If so, which illness? How does this happen?

Supplementary Table S2: Results of pairwise permanova statistics between groups based on a) Bray Curtis distance, b) Unweighted Unifrac distance and c) Weighted Unifrac distance

a) Bray Curtis distance

Group 1	Group 2	Sample size	Permutations	Sums of squares	F. Model	R^2	p-value	Corrected p-value
**Cameroon Chimpanzees**	Cameroon Gorillas	21	5000	0.16	168.04	0.90	< 0.001	0.002
**Cameroon Chimpanzees**	Zoo Chimpanzees	12	5000	0.04	77.56	0.88	0.002	0.005
**Cameroon Chimpanzees**	Zoo Gorillas	12	5000	0.04	60.04	0.86	0.002	0.005
**Cameroon Gorillas**	Zoo Chimpanzees	21	5000	0.19	208.28	0.92	< 0.001	0.001
**Cameroon Gorillas**	Zoo Gorillas	21	5000	0.18	181.3	0.91	< 0.001	0.001
**Zoo Chimpanzees**	Zoo Gorillas	12	5000	0.02	32.2	0.76	0.002	0.005

b) Unweighted Unifrac distance

Group 1	Group 2	Sample size	Permutations	Sums of squares	F. Model	R^2	p-value	Corrected p-value
**Cameroon Chimpanzees**	Cameroon Gorillas	21	5000	0.12	19.99	0.51	< 0.001	0.002
**Cameroon Chimpanzees**	Zoo Chimpanzees	12	5000	0.06	9.32	0.48	0.003	0.005
**Cameroon Chimpanzees**	Zoo Gorillas	12	5000	0.06	9.29	0.48	0.001	0.004
**Cameroon Gorillas**	Zoo Chimpanzees	21	5000	0.09	21.41	0.53	< 0.001	0.001
**Cameroon Gorillas**	Zoo Gorillas	21	5000	0.07	15.75	0.45	< 0.001	0.001
**Zoo Chimpanzees**	Zoo Gorillas	12	5000	0.02	6.51	0.39	0.003	0.005

c) Weigthed Unifrac distance

Group 1	Group 2	Sample size	Permutations	Sums of squares	F. Model	R^2	p-value	corrected p-value
**Cameroon Chimpanzees**	Cameroon Gorillas	21	5000	0.06	29.28	0.61	< 0.001	0.001
**Cameroon Chimpanzees**	Zoo Chimpanzees	12	5000	0.04	21.05	0.68	0.002	0.004
**Cameroon Chimpanzees**	Zoo Gorillas	12	5000	0.05	32.29	0.76	0.001	0.004
**Cameroon Gorillas**	Zoo Chimpanzees	21	5000	0.12	65.95	0.78	< 0.001	0.002
**Cameroon Gorillas**	Zoo Gorillas	21	5000	0.14	84.45	0.82	< 0.001	0.002
**Zoo Chimpanzees**	Zoo Gorillas	12	5000	0.01	9.95	0.50	0.002	0.004

Supplementary Table S3: Percentage of analyzed reads assigned to a known taxa for each taxonomic level

Group	Phylum	Class	Order	Family	Genus
**Cameroon Chimpanzees**	100.0	100.0	99.8	96.0	81.7
**Cameroon Gorillas**	100.0	100.0	99.9	94.8	71.1
**Zoo Chimpanzees**	100.0	99.5	99.5	95.8	72.9
**Zoo Gorillas**	100.0	99.7	99.7	91.6	65.5
**Cameroon**	100.0	100.0	99.9	95.1	74.1
**Zoo**	100.0	99.6	99.6	93.7	69.2
**Chimpanzees**	100.0	99.8	99.7	95.9	77.3
**Gorillas**	100.0	99.9	99.9	93.9	69.5
**Total**	100.0	99.9	99.8	94.6	72.3

Supplementary Figure S1: Mean taxonomic composition (relative abundance) of samples by group, at phyla level

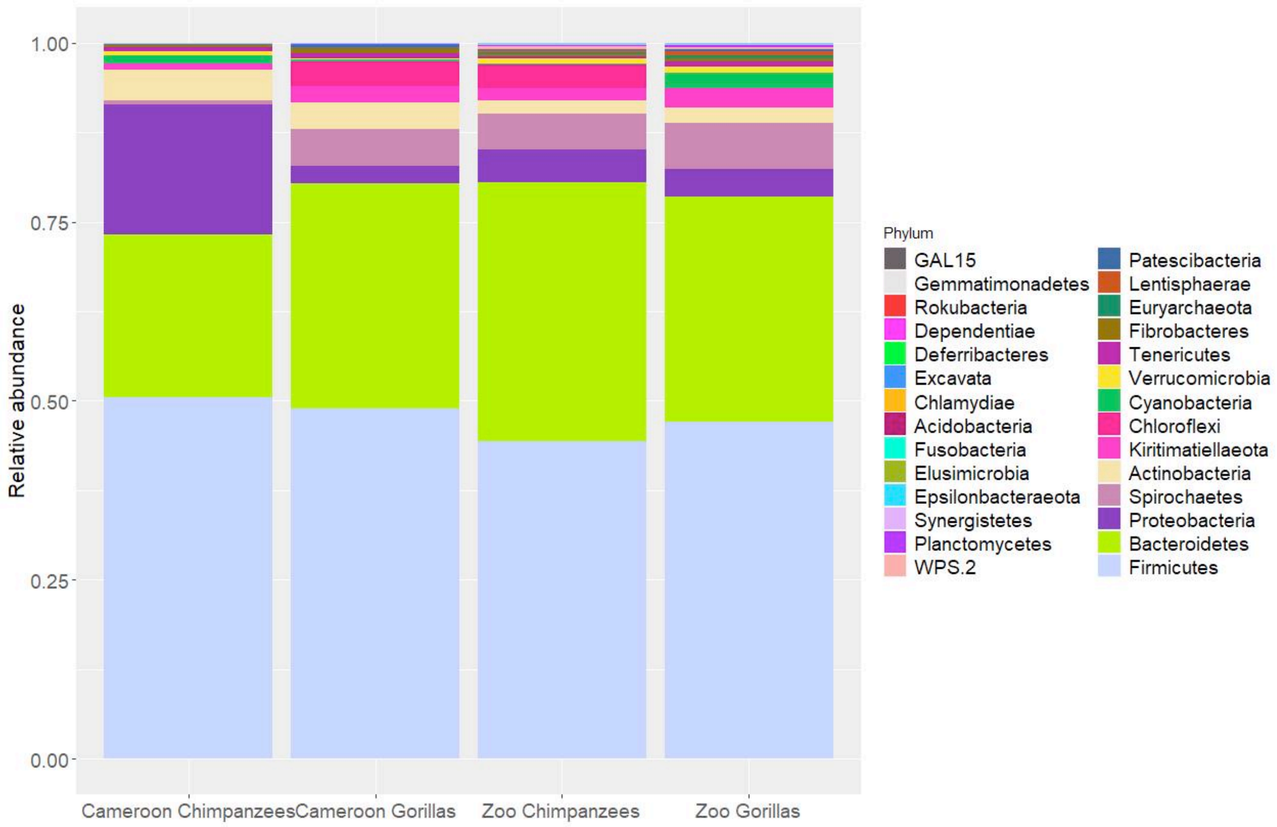

Supplementary Table S4: Mean and Standard Deviation of phyla with significant differences of relative abundance between zoo and Cameroon forest for chimpanzees and gorillas. Pairwise wilcoxon tests have been performed with Bonferroni correction. $N_{Cameroon\ chimpanzee}$=6; $N_{Zoochimpanzee}$=6; $N_{Cameroon\ gorilla}$=15; $N_{Cameroon\ gorilla}$=6.

Species	Phylum	Mean Cameroon	Mean Zoo	Adjusted p value
**Chimpanzee**	Bacteroidetes	45636 ± SD=4855	72268 ± SD=9121	0.013
**Chimpanzee**	Chloroflexi	41 ± SD=69	6542 ± SD=4681	0.013
**Chimpanzee**	Cyanobacteria	2065 ± SD=818	273 ± SD=186	0.013
**Chimpanzee**	Epsilonbacteraeota	171 ± SD=84	36 ± SD=47	0.03
**Chimpanzee**	Euryarchaeota	271 ± SD=183	769 ± SD=268	0.013
**Chimpanzee**	Spirochaetes	1284 ± SD=387	10148 ± SD=4518	0.013
**Gorilla**	Chloroflexi	6969 ± SD=4329	0 ± SD=1	0.003
**Gorilla**	Cyanobacteria	569 ± SD=520	4222 ± SD=808	< 0.001
**Gorilla**	Lentisphaerae	133 ± SD=300	1106 ± SD=464	0.005
**Gorilla**	Synergistetes	0 ± SD=0	402 ± SD=427	< 0.001
**Gorilla**	Verrucomicrobia	159 ± SD=437	1860 ± SD=1255	0.01
**Gorilla**	WPS.2	2 ± SD=4	527 ± SD=403	< 0.001

Supplementary Table S5: Mean and Standard Deviation of families with significant differences of relative abundance between zoo and Cameroon forest for chimpanzees and gorillas. Pairwise wilcoxon tests have been performed with Bonferroni correction. $N_{Cameroon\ chimpanzee}$=6; $N_{Zoochimpanzee}$=6; $N_{Cameroon\ gorilla}$=15; $N_{Cameroon\ gorilla}$=6.

Species	Phylum	Famille	Mean Cameroon	Mean Zoo	Adjusted p value
**Chimpanzee**	Actinobacteria	Coriobacteriales.Incertae.Sedis	116 ± SD=98	578 ± SD=217	0.013
**Chimpanzee**	Actinobacteria	Bifidobacteriaceae	1418 ± SD=739	102 ± SD=190	0.013
**Chimpanzee**	Bacteroidetes	Paludibacteraceae	733 ± SD=618	5651 ± SD=1234	0.03
**Chimpanzee**	Bacteroidetes	Tannerellaceae	769 ± SD=320	2352 ± SD=353	0.013
**Chimpanzee**	Bacteroidetes	Weeksellaceae	3186 ± SD=5790	0 ± SD=0	0.048
**Chimpanzee**	Chloroflexi	Anaerolineaceae	41 ± SD=69	6541 ± SD=4681	0.013
**Chimpanzee**	Epsilonbacteraeota	Campylobacteraceae	156 ± SD=73	30 ± SD=40	0.049
**Chimpanzee**	Firmicutes	Clostridiaceae.1	85 ± SD=88	3447 ± SD=1964	0.013
**Chimpanzee**	Firmicutes	Acidaminococcaceae	689 ± SD=251	5239 ± SD=3795	0.026
**Chimpanzee**	Firmicutes	Planococcaceae	14219 ± SD=9665	0 ± SD=0	0.014
**Chimpanzee**	Lentisphaerae	Victivallaceae	1 ± SD=2	194 ± SD=104	0.022
**Chimpanzee**	Proteobacteria	Desulfovibrionaceae	190 ± SD=111	1667 ± SD=781	0.013
**Chimpanzee**	Proteobacteria	Enterobacteriaceae	669 ± SD=1013	1 ± SD=1	0.026
**Chimpanzee**	Proteobacteria	Moraxellaceae	26099 ± SD=20183	0 ± SD=0	0.014
**Chimpanzee**	Spirochaetes	Spirochaetaceae	1284 ± SD=386	10145 ± SD=4520	0.013
**Gorilla**	Actinobacteria	Coriobacteriales.Incertae.Sedis	550 ± SD=316	141 ± SD=61	0.006
**Gorilla**	Actinobacteria	Eggerthellaceae	3433 ± SD=2812	765 ± SD=340	0.007
**Gorilla**	Bacteroidetes	Bacteroidales.UCG.001	0 ± SD=0	1368 ± SD=1502	< 0.001
**Gorilla**	Bacteroidetes	F082	0 ± SD=0	10423 ± SD=5114	< 0.001
**Gorilla**	Bacteroidetes	Rikenellaceae	12022 ± SD=6775	4632 ± SD=1752	0.007
**Gorilla**	Bacteroidetes	Tannerellaceae	441 ± SD=306	1847 ± SD=875	< 0.001
**Gorilla**	Chloroflexi	Anaerolineaceae	6965 ± SD=4330	0 ± SD=1	0.003
**Gorilla**	Firmicutes	Clostridiales.vadinBB60.group	3573 ± SD=2727	8713 ± SD=1705	0.007

**Gorilla**	Firmicutes	Family.XIII	5365 ± SD=1887	1069 ± SD=336	< 0.001
**Gorilla**	Firmicutes	Lachnospiraceae	27560 ± SD=6688	16590 ± SD=3001	0.004
**Gorilla**	Lentisphaerae	Victivallaceae	0 ± SD=0	488 ± SD=573	< 0.001
**Gorilla**	Synergistetes	Synergistaceae	0 ± SD=0	402 ± SD=427	< 0.001
**Gorilla**	Tenericutes	Acholeplasmataceae	384 ± SD=400	0 ± SD=0	0.039
**Gorilla**	Verrucomicrobia	Puniceicoccaceae	150 ± SD=429	1860 ± SD=1255	0.01

Supplementary Table S6: Mean and Standard Deviation of genera with significant differences of relative abundance between zoo and Cameroon forest for chimpanzees and gorillas. Pairwise wilcoxon tests have been performed with Bonferroni correction. $N_{Cameroon\ chimpanzee}$=6; $N_{Zoochimpanzee}$=6; $N_{Cameroon\ gorilla}$=15; $N_{Cameroon\ gorilla}$=6.

Species	Phylum	Famille	Genre	Mean Cameroon	Mean Zoo	Adjusted p value
**Chimpanzee**	Actinobacteria	Coriobacteriales Incertae Sedis	Phoenicibacter	79 ± SD=62	576 ± SD=216	0.013
**Chimpanzee**	Actinobacteria	Bifidobacteriaceae	Bifidobacterium	1418 ± SD=739	102 ± SD=190	0.013
**Chimpanzee**	Actinobacteria	Atopobiaceae	Olsenella	777 ± SD=510	0 ± SD=0	0.017
**Chimpanzee**	Bacteroidetes	Prevotellaceae	Prevotella 2	0 ± SD=0	1431 ± SD=1030	0.017
**Chimpanzee**	Bacteroidetes	Rikenellaceae	dgA 11 gut group	17 ± SD=29	707 ± SD=350	0.03
**Chimpanzee**	Bacteroidetes	Prevotellaceae	Prevotella 1	62 ± SD=105	773 ± SD=347	0.049
**Chimpanzee**	Bacteroidetes	Prevotellaceae	Prevotellaceae UCG 001	1815 ± SD=802	10295 ± SD=5570	0.013
**Chimpanzee**	Bacteroidetes	Tannerellaceae	Parabacteroides	526 ± SD=311	2352 ± SD=353	0.013
**Chimpanzee**	Bacteroidetes	Prevotellaceae	Prevotella 9	8255 ± SD=3449	16131 ± SD=4640	0.026
**Chimpanzee**	Bacteroidetes	Prevotellaceae	Prevotella 7	10565 ± SD=4126	0 ± SD=1	0.022
**Chimpanzee**	Bacteroidetes	Prevotellaceae	Prevotellaceae NK3B31 group	1173 ± SD=1743	0 ± SD=0	0.017
**Chimpanzee**	Bacteroidetes	Muribaculaceae	metagenome	708 ± SD=818	0 ± SD=0	0.017
**Chimpanzee**	Bacteroidetes	Flavobacteriaceae	Flavobacterium	1129 ± SD=2658	0 ± SD=0	0.048
**Chimpanzee**	Bacteroidetes	Weeksellaceae	Empedobacter	595 ± SD=1436	0 ± SD=0	0.029
**Chimpanzee**	Chloroflexi	Anaerolineaceae	Flexilinea	41 ± SD=69	6541 ± SD=4681	0.013
**Chimpanzee**	Epsilonbacteraeota	Campylobacteraceae	Campylobacter	156 ± SD=73	30 ± SD=40	0.049
**Chimpanzee**	Euryarchaeota	Methanomethylophilaceae	Candidatus Methanomethylophilus	0 ± SD=0	59 ± SD=58	0.017
**Chimpanzee**	Firmicutes	Clostridiaceae 1	Clostridium sensu stricto 1	0 ± SD=0	1724 ± SD=2133	0.017
**Chimpanzee**	Firmicutes	Ruminococcaceae	Ruminiclostridium 6	10 ± SD=23	586 ± SD=707	0.037
**Chimpanzee**	Firmicutes	Clostridiaceae 1	Sarcina	85 ± SD=88	1721 ± SD=1103	0.013

**Chimpanzee**	Firmicutes	Lachnospiraceae	Acetitomaculum	70 ± SD=22	1271 ± SD=848	0.013
**Chimpanzee**	Firmicutes	Lachnospiraceae	Ruminococcus gauvreauii group	43 ± SD=34	522 ± SD=508	0.03
**Chimpanzee**	Firmicutes	Ruminococcaceae	Ruminococcus 1	202 ± SD=92	2163 ± SD=1846	0.013
**Chimpanzee**	Firmicutes	Acidaminococcaceae	Phascolarctobacterium	689 ± SD=251	5239 ± SD=3795	0.026
**Chimpanzee**	Firmicutes	Clostridiales vadinBB60 group	gut metagenome	194 ± SD=121	669 ± SD=258	0.013
**Chimpanzee**	Firmicutes	Lachnospiraceae	Roseburia	1310 ± SD=580	368 ± SD=223	0.013
**Chimpanzee**	Firmicutes	Ruminococcaceae	Butyricicoccus	233 ± SD=168	45 ± SD=21	0.013
**Chimpanzee**	Firmicutes	Lachnospiraceae	Lachnoclostridium	1532 ± SD=392	271 ± SD=160	0.013
**Chimpanzee**	Firmicutes	Lachnospiraceae	Oribacterium	7104 ± SD=1197	887 ± SD=532	0.013
**Chimpanzee**	Firmicutes	Ruminococcaceae	Ruminococcaceae UCG 008	6492 ± SD=4439	804 ± SD=605	0.026
**Chimpanzee**	Firmicutes	Family XIII	Family XIII UCG 001	440 ± SD=223	46 ± SD=13	0.03
**Chimpanzee**	Firmicutes	Lachnospiraceae	Lachnospiraceae UCG 004	1792 ± SD=610	179 ± SD=105	0.013
**Chimpanzee**	Firmicutes	Lachnospiraceae	Anaerostipes	446 ± SD=202	36 ± SD=32	0.013
**Chimpanzee**	Firmicutes	Ruminococcaceae	Ruminiclostridium 5	1307 ± SD=587	86 ± SD=88	0.013
**Chimpanzee**	Firmicutes	Lachnospiraceae	GCA 900066575	514 ± SD=259	28 ± SD=25	0.03
**Chimpanzee**	Firmicutes	Planococcaceae	Rummeliibacillus	960 ± SD=1068	0 ± SD=0	0.008
**Chimpanzee**	Firmicutes	Planococcaceae	Kurthia	8568 ± SD=8385	0 ± SD=0	0.008
**Chimpanzee**	Proteobacteria	Desulfovibrionaceae	Desulfovibrio	190 ± SD=111	1556 ± SD=745	0.013
**Chimpanzee**	Proteobacteria	Burkholderiaceae	Comamonas	1737 ± SD=4172	0 ± SD=0	0.048
**Chimpanzee**	Proteobacteria	Moraxellaceae	Acinetobacter	26099 ± SD=20183	0 ± SD=0	0.014
**Chimpanzee**	Spirochaetes	Spirochaetaceae	Sphaerochaeta	284 ± SD=221	2452 ± SD=1158	0.013
**Chimpanzee**	Spirochaetes	Spirochaetaceae	Treponema 2	1000 ± SD=207	7670 ± SD=5015	0.013
**Gorilla**	Actinobacteria	Atopobiaceae	Libanicoccus	718 ± SD=789	0 ± SD=0	0.003
**Gorilla**	Actinobacteria	Eggerthellaceae	Senegalimassilia	777 ± SD=752	66 ± SD=94	0.012
**Gorilla**	Bacteroidetes	Prevotellaceae	Prevotella 1	89 ± SD=129	3277 ± SD=2944	0.003
**Gorilla**	Bacteroidetes	Prevotellaceae	Prevotellaceae UCG 001	2063 ± SD=1823	5416 ± SD=2194	0.028
**Gorilla**	Bacteroidetes	Prevotellaceae	Prevotellaceae UCG 003	482 ± SD=326	2051 ± SD=940	0.004
**Gorilla**	Bacteroidetes	Prevotellaceae	Prevotellaceae UCG 004	2393 ± SD=2692	186 ± SD=171	< 0.001

**Gorilla**	Bacteroidetes	Rikenellaceae	dgA 11 gut group	0 ± SD=0	491 ± SD=382	< 0.001
**Gorilla**	Bacteroidetes	Rikenellaceae	Rikenellaceae RC9 gut group	12000 ± SD=6776	3904 ± SD=1836	0.007
**Gorilla**	Bacteroidetes	Tannerellaceae	Parabacteroides	439 ± SD=304	1847 ± SD=875	< 0.001
**Gorilla**	Chloroflexi	Anaerolineaceae	Flexilinea	6965 ± SD=4330	0 ± SD=1	0.003
**Gorilla**	Euryarchaeota	Methanomethylophilaceae	Candidatus Methanomethylophilus	361 ± SD=321	13 ± SD=18	0.003
**Gorilla**	Firmicutes	Clostridiaceae 1	Clostridium sensu stricto 1	28 ± SD=99	322 ± SD=436	0.026
**Gorilla**	Firmicutes	Family XIII	Family XIII AD3011 group	4185 ± SD=1520	313 ± SD=46	< 0.001
**Gorilla**	Firmicutes	Lachnospiraceae	Eubacterium eligens group	2 ± SD=6	107 ± SD=68	< 0.001
**Gorilla**	Firmicutes	Lachnospiraceae	Eubacterium hallii group	1284 ± SD=808	73 ± SD=75	0.003
**Gorilla**	Firmicutes	Lachnospiraceae	Eubacterium ruminantium group	46 ± SD=63	482 ± SD=447	0.04
**Gorilla**	Firmicutes	Lachnospiraceae	Acetitomaculum	392 ± SD=339	50 ± SD=36	0.006
**Gorilla**	Firmicutes	Lachnospiraceae	Agathobacter	652 ± SD=578	46 ± SD=61	0.01
**Gorilla**	Firmicutes	Lachnospiraceae	GCA 900066575	0 ± SD=0	81 ± SD=75	< 0.001
**Gorilla**	Firmicutes	Lachnospiraceae	Lachnospiraceae ND3007 group	4202 ± SD=3244	94 ± SD=88	< 0.001
**Gorilla**	Firmicutes	Lachnospiraceae	Lachnospiraceae NK3A20 group	704 ± SD=442	101 ± SD=226	0.016
**Gorilla**	Firmicutes	Lachnospiraceae	Lachnospiraceae NK4A136 group	18 ± SD=48	724 ± SD=561	0.001
**Gorilla**	Firmicutes	Lachnospiraceae	Lachnospiraceae XPB1014 group	0 ± SD=0	3992 ± SD=2363	< 0.001
**Gorilla**	Firmicutes	Lachnospiraceae	Pseudobutyrivibrio	4267 ± SD=2037	37 ± SD=26	< 0.001
**Gorilla**	Firmicutes	Ruminococcaceae	Eubacterium coprostanoligenes group	12311 ± SD=4829	4785 ± SD=2319	0.004
**Gorilla**	Firmicutes	Ruminococcaceae	Candidatus Soleaferrea	91 ± SD=78	701 ± SD=293	0.006
**Gorilla**	Firmicutes	Ruminococcaceae	Hydrogenoanaerobacterium	640 ± SD=287	64 ± SD=41	< 0.001
**Gorilla**	Firmicutes	Ruminococcaceae	Oscillibacter	124 ± SD=99	370 ± SD=107	0.016
**Gorilla**	Firmicutes	Ruminococcaceae	Ruminiclostridium 5	1954 ± SD=1797	70 ± SD=48	< 0.001
**Gorilla**	Firmicutes	Ruminococcaceae	Ruminiclostridium 6	12 ± SD=30	962 ± SD=621	0.001
**Gorilla**	Firmicutes	Ruminococcaceae	Ruminococcaceae UCG 002	1175 ± SD=517	4267 ± SD=1613	< 0.001
**Gorilla**	Firmicutes	Ruminococcaceae	Ruminococcaceae UCG 003	29 ± SD=62	432 ± SD=351	0.006
**Gorilla**	Firmicutes	Ruminococcaceae	Ruminococcaceae UCG 005	2970 ± SD=1196	5665 ± SD=1671	0.02
**Gorilla**	Firmicutes	Ruminococcaceae	Ruminococcaceae UCG 013	177 ± SD=191	1017 ± SD=379	< 0.001

**Gorilla**	Firmicutes	Erysipelotrichaceae	Erysipelotrichaceae UCG 006	0 ± SD=0	38 ± SD=41	< 0.001
**Gorilla**	Firmicutes	Erysipelotrichaceae	Holdemanella	307 ± SD=316	10 ± SD=15	0.003
**Gorilla**	Firmicutes	Erysipelotrichaceae	Ileibacterium	0 ± SD=0	773 ± SD=1777	0.002
**Gorilla**	Firmicutes	Erysipelotrichaceae	Solobacterium	1241 ± SD=869	209 ± SD=350	0.01
**Gorilla**	Proteobacteria	uncultured	gut metagenome	3 ± SD=12	216 ± SD=165	< 0.001
**Gorilla**	Proteobacteria	Succinivibrionaceae	Ruminobacter	1 ± SD=3	601 ± SD=248	< 0.001
**Gorilla**	Proteobacteria	Burkholderiaceae	Parasutterella	0 ± SD=0	2458 ± SD=3104	< 0.001
**Gorilla**	Spirochaetes	Spirochaetaceae	Sphaerochaeta	796 ± SD=505	5838 ± SD=3142	< 0.001
**Gorilla**	Tenericutes	Acholeplasmataceae	Acholeplasma	384 ± SD=400	0 ± SD=0	0.039
**Gorilla**	Verrucomicrobia	Puniceicoccaceae	Cerasicoccus	112 ± SD=430	1441 ± SD=1235	0.003
